# Constructing an evaluation framework for primary pharmacovigilance systems in developing countries: a case study from China

**DOI:** 10.3389/fphar.2025.1657574

**Published:** 2025-11-18

**Authors:** Shuang Lei, Shuzhi Lin, Xiaoying Zhu, Wei Liu, Qian Liu, Lin Yin, Bianling Feng

**Affiliations:** 1 The Department of Pharmacy Administration, School of Pharmacy, Xi’an Jiaotong University, Xi’an, Shaanxi, China; 2 Department of Pharmacy, Norinco General Hospital, Xi’an, Shaanxi, China

**Keywords:** pharmacovigilance system, developing country, evaluation indicator framework, delphi method, analytic hierarchy process (AHP)

## Abstract

**Objective:**

This study aimed to develop an evaluation framework for primary pharmacovigilance systems based on the Chinese context, with potential applicability to regions with similar system maturity. The framework was informed by the WHO manual and tailored to national policies and resource constraints to support consistent pharmacovigilance practices in similar settings and enhance medication safety.

**Methods:**

This study employed semi-structured interviews, literature review, brainstorming, group discussions, and other common methods to establish a preliminary indicator system. The Delphi Method was employed to screen indicators at various levels based on inclusion principles. Subsequently, the Analytic Hierarchy Process (AHP) was applied to determine indicator weights.

**Results:**

The effective response rates for both rounds of questionnaires were 100%. Furthermore, the coefficients of reliability were 0.87 and 0.88 for each round, and the Kendall’s W for the process and outcome indicators were 0.173 and 0.236. The significance tests for Kendall’s W were both P < 0.001. The final evaluation indicator system consisted of 3 first-level indicators, 9 second-level indicators, and 35 third-level indicators.

**Conclusion:**

This study developed a practical and context-sensitive evaluation framework for primary pharmacovigilance systems in China. The framework offers a feasible assessment tool and may serve as a reference for regions with similar development stages or incomplete pharmacovigilance systems, providing guidance for system improvement and resource allocation.

## 1 Introduction

Drug safety is a significant worldwide public health issue. Even in the wake of the thalidomide tragedy of the 1960s, when many governments established pharmacovigilance systems to ensure the quality, safety, and effectiveness of various drugs approved according to the guidelines of the World Health Organization (WHO) (Garashi et al., 2021). Meanwhile, China also recognized the importance of establishing a systematic mechanism for drug safety regulation. In 1989, the Ministry of Health established the National Center for Adverse Drug Reaction Monitoring as a specialized technical institution and initiated pilot programs for adverse drug reaction (ADR) reporting in more than ten medical institutions nationwide. By 2002, ADR monitoring centers had been established in all 31 provinces, autonomous regions, and municipalities, gradually extending to prefecture- and county-level units. The number of reporting institutions steadily increased, forming a nationwide ADR monitoring network that laid a solid foundation for the development of China’s pharmacovigilance system. In 2001, the Drug Administration Law explicitly stipulated that “the state shall implement an adverse drug reaction reporting system,” marking the formal legalization of China’s ADR monitoring efforts. Pharmacovigilance is the discovery, evaluation, understanding, and prevention of adverse drug reactions (ADR) and any other issues related to drugs. The construction pharmacovigilance systems enable the comprehensive monitoring of the safety of drugs throughout their development and use and allows for timely adjustments based on the latest information.

On a global scale, there are significant disparities among countries in the development and implementation of pharmacovigilance systems. Although most developed countries have sophisticated systems, many developing countries still lack adequately functioning systems (Hussain and Hassali, 2019; Olsson et al., 2015; Tiemersma et al., 2021). For these developing countries, the common challenges they face include insufficient reporting of adverse reactions, shortages of human resources, inadequate financial resources, and weaknesses in political and legal frameworks (Garashi et al., 2021; Hussain and Hassali, 2019; Isah et al., 2012; Suwankesawong et al., 2016; Menang et al., 2023). China in particular is in the process of actively developing pharmacovigilance systems to align with international standards. In the “Guidelines for Pharmacovigilance Quality Management” (GVP) released at the end of 2021 (National Medical Products Administration, 2021), China’s government proposed to establish a pharmacovigilance system. Currently, China’s pharmacovigilance technical support system relies primarily on a three-level monitoring network for adverse drug reactions (national, provincial, and municipal/prefectural -level drug adverse reaction monitoring centers) that performs mostly passive monitoring (Ren et al., 2021; Zhu, 2008; Shao and Tang, 2014). To collect drug safety data more comprehensively and apply it to clinical practice under limited resource conditions, different levels of institutions in China have different functions. For example, the municipal pharmacovigilance center plays a key role in the collection and evaluation of adverse drug reaction/adverse drug event (ADR/ADE) reports, and provincial and national centers integrate information collected at the municipal level and implement preventive measures as necessary.

Similar to many developing countries, China’s pharmacovigilance policies and legal frameworks are relatively weak, and the development of its pharmacovigilance system is still in the early stages. Particularly for municipal level pharmacovigilance centers, there are challenges such as unclear institutional construction, organizational structure confusion, insufficient professional personnel, and chaotic work systems. These challenges hinder the analysis of ADR/ADE-related data and the identification of potential drug safety issues and underscoring the importance of evaluating China’s pharmacovigilance system itself. To assess pharmacovigilance systems using reliable indicators, the World Health Organization (WHO) has issued “WHO Pharmacovigilance Indicators: A Practical Manual for the Assessment of Pharmacovigilance Systems” (hereinafter referred to as the “WHO manual”) (World Health Organization, 2015), which provides a framework and indicators for pharmacovigilance assessment.

Currently, most studies of pharmacovigilance systems focus on evaluation using WHO indicators. However, the indicators in the WHO manual may not be directly applicable in many countries. Research utilizing WHO manual indicators has run into challenges in obtaining data for these indicators. For instance, a lack of professional pharmacovigilance personnel can impact the acquisition of process indicators. Additionally, limitations in structural indicators may not fully describe the true functionality of a country’s pharmacovigilance system. This suggests that the WHO manual indicator system may not be suitable for evaluating immature pharmacovigilance systems, especially in developing countries at the early stages of pharmacovigilance development (Fenfang et al., 2021; Qato, 2018; Opadeyi et al., 2018). Directly evaluating the performance results of drug risk management and investigating issues such as drug abuse and medication errors may be challenging for such countries. This is because many of these countries, much like China’s municipal level pharmacovigilance centers, are still only at the adverse drug reaction monitoring stage of development, and a transitional approach to system evaluation is needed based on specific domestic policy conditions and resource limitations. However, there is currently no unified indicator framework for a comprehensive evaluation of the performance of primary pharmacovigilance systems. There is also limited research that comprehensively considers the pharmacovigilance process, outcomes, and alignment of national policies with regional resource constraints within a single pharmacovigilance evaluation system.

Therefore, in this study we reference the requirements of the WHO manual and consider national policy development and regional resource constraints in order to propose just such a comprehensive evaluation method. Taking the Chinese municipal level pharmacovigilance centers as an example, we construct a primary pharmacovigilance system evaluation indicator framework for developing countries that face similar challenges as China. We hope that this approach will result in a pharmacovigilance system assessment tool that better aligns with the needs of developing regions and contributes to the improvement of global drug safety standards.

## 2 Materials and methods

### 2.1 Study design

This study employed semi-structured interviews, literature review, brainstorming, group discussions, and other common methods to establish a preliminary indicator system. The preliminary framework was developed around the Donabedian three-dimensional theory. Based on this framework, the Delphi Method was used to refine and screen indicators at each level according to inclusion criteria. Additionally, the Analytic Hierarchy Process (AHP) was applied to determine the weight of each indicator.

The Donabedian three-dimensional theory, originally designed for evaluating healthcare quality, provides a structured framework of structure, process, and outcome. It supports comprehensive assessments, identifies system weaknesses, and guides improvements. Emphasizing interrelations among dimensions, its application varies by context; resource-limited settings may prioritize structure, while efficiency-focused contexts target process. Widely adopted globally, the model informs healthcare quality management, health services research, indicator development, and policy-making, offering a holistic view of system development.

The Delphi method, also known as the expert survey method, is widely used in various fields such as business, the military, education, and healthcare. It involves soliciting expert opinions through multiple rounds of anonymous feedback (Nasa et al., 2021; Niederberger and Spranger, 2020) and is therefore considered to be an efficient method for judgment and prediction that produces results that are widely representative and reliable. AHP is a multi-criteria decision analysis method that decomposes problems into a hierarchical structure that can aid decision-makers in better understanding the complexity of an issue and balancing and deciding among multiple complex options (Wu et al., 2022). Although the Delphi method focuses on gathering expert opinions and reaching consensus through multiple rounds of feedback, AHP focuses on synthesizing and quantifying diverse expert opinions in order to facilitating a more comprehensive comparison between and possibly integration of different perspectives.

### 2.2 Sampling and recruitment

At the outset of this study we first pre-investigated the applicability of preliminarily constructed indicators, and selected five municipal-level cities, Xi’an, Baoji, Hanzhong, Xianyang, and Weinan in China, for pre-investigation, taking into account factors such as regional economy, completeness of pharmacovigilance system construction, and system implementation. Together, these cities account for 50% of the prefecture-level cities in the province, ensuring both strong representativeness and broad applicability of the findings. Eligible participants included Drug Administration employees or principals directly involved in the implementation of pharmacovigilance policies. Sampling was purposeful: the head of a given provincial pharmacovigilance department emailed a study information sheet to all potential participants and asked them to contact the researchers for more information.

Subsequently, the established indicators were provided to experts in the field of pharmacovigilance from the Drug Regulatory Authority, medical institutions, and universities for further research. During the recruitment phase, potential experts were contacted via telephone, and detailed explanations about the research and its objectives were discussed. Those expressing interest and volunteering to participate were asked to read and complete informed consent documents, and the strictest confidentiality was maintained. Ethical approval for this study was provided by the Ethics Committee of the Medical Department of Xi’an Jiaotong University (No. 2023–2,361).

### 2.3 Data collection

#### 2.3.1 Selection of experts

A Purposeful sampling method was employed to select individuals to serve on the Delphi expert panel. The criteria for expert qualification were as follows.

1. This study spans relevant disciplines such as pharmacovigilance, drug regulation, public health, and pharmaceutical administration to ensure fairness and comprehensiveness in the evaluation outcomes.
2. Experts are required to have a minimum of 5 years of experience in theoretical research or practical management within the field of pharmacovigilance. They should possess extensive practical experience in areas such as drug safety monitoring, pharmaceutical administration, and risk management.
3. A bachelor’s degree or higher, interest in the study, and voluntary participation.

#### 2.3.2 Instruments

**FIGURE 1:**
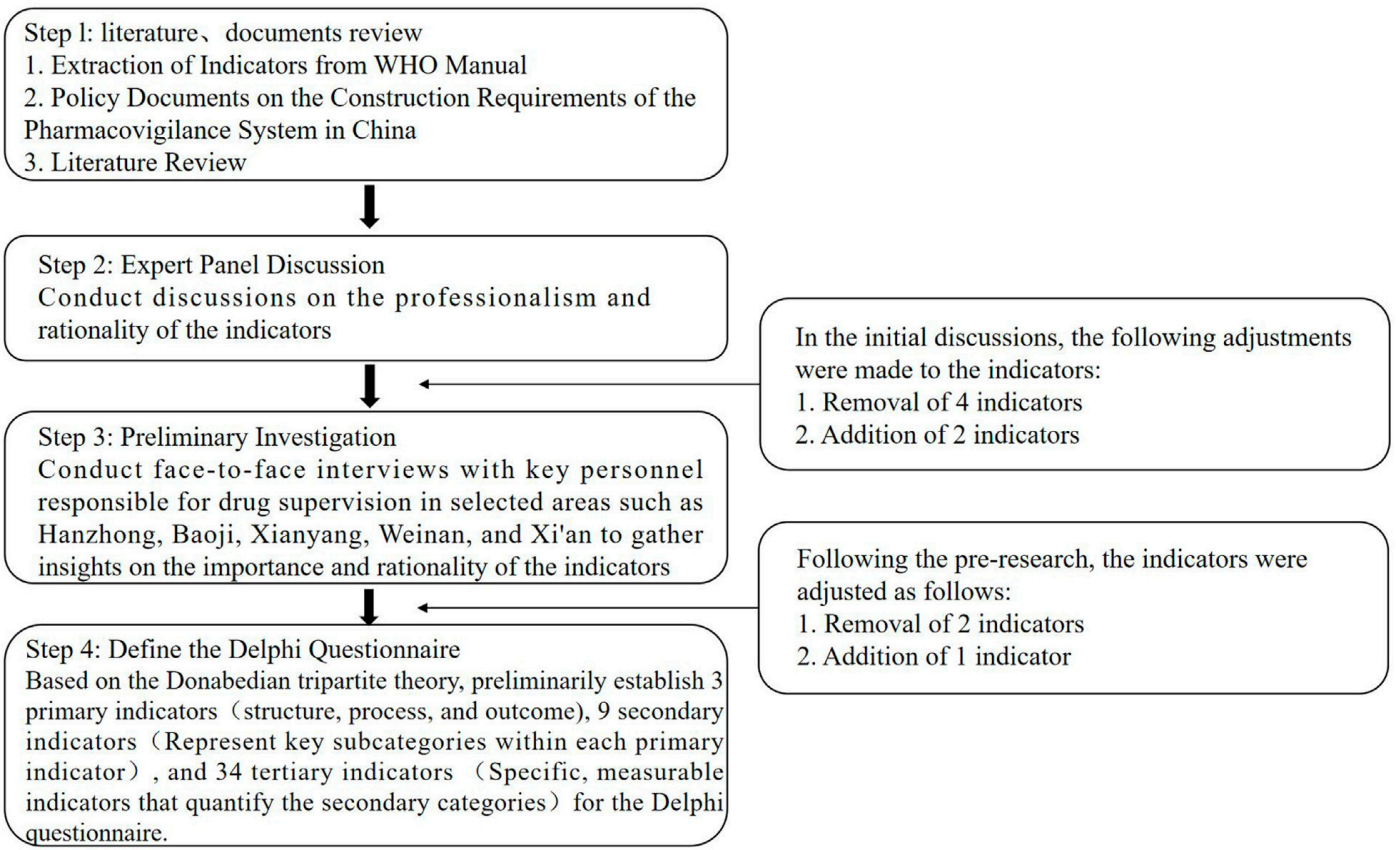
The development process for the Delphi Survey Questionnaire.

The development process of the main Delphi questionnaire is illustrated in Figure 1. First, we summarized the framework and basic indicator requirements for pharmacovigilance system construction based on the WHO manual and combined this with a detailed framework formulated through a review of China’s historical policies on adverse drug reaction regulation and literature research. Second, we conducted interviews with four experts from provincial drug regulatory authorities based on the preliminary indicators, making initial modifications that focused on the indicators’ professional relevance, reasonability, and scope. Third, we conducted pre-tests and interviews with key personnel from drug regulatory agencies in Hanzhong, Baoji, Xianyang, Weinan, and Xi’an, that focused on the importance and reasonability of the indicators and made further modifications to them based on these interview results. Finally, the Delphi questionnaire, was revised for appropriate wording and was then used as the survey tool to obtain expert responses.

The Delphi survey questionnaire (Supplementary File 1) for evaluating municipal-level city pharmacovigilance systems was based on Donabedian’s three-dimensional theory. The preliminary framework consisted of three primary indicators corresponding to the dimensions of the SPO model—Structure, Process, and Outcome—nine secondary indicators representing key subcategories within each primary indicator (e.g., Organization, Resource Inputs, and Staffing under Structure), and 34 tertiary indicators offering specific, measurable metrics for the secondary categories (e.g., Financial Inputs under Resource Inputs).

The questionnaire consisted of three main sections: The first section collected experts’ demographic and professional background information. The second section presented the complete list of candidate indicators, each accompanied by a clear operational definition (see Supplementary File 2; Supplementary Tables S1–S3). Experts were asked to assess the importance of each indicator on a 5-point Likert scale in the following order: 5 = very important; 4 = quite important; 3 = moderately important; 2 = not very important; and 1 = not important. The final section included an open-ended comment field beneath each indicator, allowing experts to propose deletions, modifications, or additions of indicators, with a requirement to provide justification for any suggested changes.

Two rounds of questionnaire consultations were then conducted via email. In the first round, the questionnaire content included the research objectives, expert profiles, indicator explanations, and a section for opinions on modifications or deletions to gather expert feedback. The second round of consultations involved minor adjustments based on the statistical results of the first round. After two rounds of consultations, consensus was deemed to be reached. Following the consensus on the indicators, we utilized the AHP to determine the weights of each indicator.

### 2.4 Data analysis

#### 2.4.1 Selection criteria of indicators

Indicators were retained based on specific criteria. That is, the Delphi indicators were included only if they simultaneously met the conditions in Table 1.

**TABLE 1:** Delphi indicator inclusion criteria.

Indicator criteria	Interpretation
Coefficient of junction Cj > 70%	The coefficient of junction indicates the level of interest that experts have in the research project. It is typically assessed using the survey response rate, with a response rate of 70% or above generally considered indicative of good expert engagement
Coefficient of reliability Cr ≥ 0.7	The calculation of expert recommendation value assessment is performed using the formula Cr = (Ca + Cs)/2, where Ca is the coefficient of adjudication, reflecting the extent to which an expert’s judgment is based on multiple reliable sources, including theoretical analysis, practical experience, peer knowledge, and intuition; and Cs is the expert familiarity score, quantifying the expert’s self-assessed familiarity with each indicator on a five-point Likert scale ranging from “very familiar” (1) to “not familiar” (0). Generally, a Cr ≥ 0.7 indicates a high level of expert authority and ensures that the expert opinions have substantial research value
Kendall’s coefficient of concordance (Kendall’s W)	Kendall’s W is used to reflect whether expert opinions obtained are consistent. Kendall’s W ranges from 0 to 1, where 0 indicates no concordance (ratings are random) and 1 indicates perfect concordance (all experts judge the items identically). Values between these extremes reflect varying degrees of agreement. Generally, a Kendall’s W < 0.2 is considered to indicate poor agreement. At the same time, if the Kendall’s W has a p < 0.001, it means that the result of the emergence of coordination of expert opinions is plausible
Coefficient of variation CV < 0.25	The coefficient of variation reflects whether there is a large disagreement in the evaluation of each indicator by the experts. If CV > 0.25, then there is not enough coordination of experts for that indicator
Mean ≥4	The higher the value of the mean of all experts’ importance ratings for a given indicator, the greater its importance, and in this study, the mean value of ≥4 was determined as the inclusion criterion after expert consultation

#### 2.4.2 Statistical analysis

The consultation data was input into Microsoft Excel software, and statistical analysis of the results was conducted using IBM SPSS Statistics for Windows version 25.0. The methods included descriptive statistics and tests such as Kendall’s W, where p < 0.001 was considered to indicate a statistically significant result.

The Analytic Hierarchy Process was performed using Yaahp 10.3 software to construct a hierarchical model and calculate both initial and normalized weights for each level of indicators. Following the completion of the Delphi rounds, experts were invited to conduct pairwise comparisons of the indicators at the same level based on Saaty’s 1–9 scale via a separate, structured questionnaire, thereby forming judgment matrices. In this scale, a score of 1 indicates that both indicators are equally important, and a score of 9 indicates that one indicator is extremely more important than the other. The resulting judgment matrices were evaluated for logical consistency using the Consistency Ratio (CR), calculated as CR = CI/RI < 0.1, where CI = (λ_max_–n)/(n – 1), λ_max_ is the maximum eigenvalue, n is the matrix order, and RI represents the average random consistency index. A matrix was considered acceptable when CR < 0.1; otherwise, experts were asked to revise their comparisons until the criterion was met. All finalized matrices satisfied the CR < 0.1 requirement. To obtain the final comprehensive weights, individual expert weight vectors were aggregated using the arithmetic mean method, yielding a consensus-based weighting system.

## 3 Results

### 3.1 Basic demographic and professional information of the experts

This study conducted two rounds of expert consultations. By the end of the second round, the inclusion criteria for indicators had been largely satisfied, no further significant new insights were expected, and expert opinions had reached a consensus. In the first round, a total of 18 experts with more than 5 years of experience in the field of pharmacovigilance from regulatory agencies, healthcare institutions, and universities participated in the Delphi process. Seventeen of these experts continued to the second round, as one expert retired. The basic demographic and professional information of the experts is shown in Table 2. The participation rate was 100% for both rounds, indicating a high level of engagement, and the effective response rate for both rounds of the questionnaire was 100%. Furthermore, the coefficients of reliability were 0.87 and 0.88 for each round, sequentially, which were both above 0.70, indicating that the experts had a high level of authority. Therefore, the results were deemed to be reliable and convincing.

**TABLE 2:** Basic demographical and professional information of the experts.

Category	One round of expert composition (*n* = 18)		Composition of experts for the second round (*n* = 17)	
	Numbers	Percentage (%)	Numbers	Percentage (%)
Age (years)				
30–39	5	27.78%	5	29.41%
40–49	7	38.89%	7	41.18%
≥50	6	33.33%	5	29.41%
Field of research				
Drug risk management	8	44.44%	7	41.18%
Pharmacovigilance	7	38.89%	7	41.18%
Pharmaceutical administration and public health	3	16.67%	3	17.65%
Experience (years)				
5–9	9	50.00%	9	52.94%
10–19	6	33.33%	5	29.41%
≥20	3	16.67%	3	17.65%
Education				
Doctoral degree	4	22.22%	4	23.53%
Master’s degree	5	27.78%	5	29.41%
Bachelor’s degree	9	50.00%	8	47.06%

### 3.2 Delphi indicator construction results

The established preliminary indicator framework with primary indicators encompassed three dimensions: structure, process, and outcomes. Within the structure indicators, there were three secondary indicators: organizational structure, resource inputs, and staffing. The process indicators’ secondary indicators revolved around the specific content and support of pharmacovigilance, including management and review, ADR/ADE reporting and monitoring, and pharmaceutical risk management and feedback, and the outcome indicators demonstrate certain performance aspects related to pharmacovigilance activities, including the effectiveness of adverse reaction monitoring, the number of regulatory actions, and the timeliness of communication and amount of feedback. Finally, each secondary indicator had corresponding tertiary indicators.

After two rounds of expert consultations, the Kendall’s W for the indicators were 0.173 and 0.236, respectively. The significance tests for Kendall’s W were both P < 0.001, indicating convergence of expert opinions, and the consistency of expert opinions in the second round was higher than in the first round. In the first consultation round, the average importance of each index ranged from 4.11 to 5.00, with a coefficient of variation between 0.00 and 0.34. Based on the Delphi expert opinions and group discussions, we revised five indicators, including the removal of “Number of recalled drugs” and “Number of changes in safety information in drug instructions” under the regulatory actions in the outcome indicators, and the addition of “Number of recognized risk signals” and “Number of training sessions for promotion.” In addition, we added “Establishment of Regulatory Framework” to the organizational structure under the structural indicators. In the second round of consultations, there was a disputed indicator, “Number of reported cases of adverse drug reaction per 1,000,000 people”. This indicator is a mandatory assessment criterion for higher-level departments to evaluate the work of municipal-level departments, however, and it was retained after some discussion. The average importance of the remaining indicators was >4, with CV < 0.25 for all of them, meeting the inclusion criteria. In the end, after two rounds of negotiations, a consensus was reached on the pharmacovigilance system evaluation indicator system that included three primary indicators, nine secondary indicators, and 35 tertiary indicators. See Table 3 for the specific details.

**TABLE 3:** Inclusion and weighting results of evaluation indicators for China’s primary drug safety surveillance system.

Indicator	First-round Delphi results		Second-round Delphi results		AHP weights
	Score (mean ± s)	CV	Score (mean ± s)	CV	
1. Structural					**0.3531**
1.1 Organization					0.0953
1.1.1 Pharmacovigilance centers/departments/units	4.94 ± 0.236	0.05	4.71 ± 0.470	0.10	0.0485
1.1.2 Advisory board oe experts	4.40 ± 0.784	0.18	4.24 ± 0.562	0.13	0.0253
1.1.3 Establishment or regulatory framework	First round of Delphi increased indicators		4.65 ± 0.606	0.13	0.0215
1.2 Resource inputs					**0.1465**
1.2.1 Financial inputs	4.72 ± 0.575	0.12	4.88 ± 0.332	0.07	0.0487
1.2.2 Pharmacovigilance information system	4.83 ± 0.514	0.11	4.59 ± 0.712	0.16	0.0348
1.2.3 Pharmacovigilance information resources	4.61 ± 0.608	0.13	4.47 ± 0.514	0.11	0.0250
1.2.4 Pharmacovigilance information completion tool	4.72 ± 0.669	0.14	4.29 ± 0.772	0.18	0.0246
1.2.5 Dissemination of pharmacovigilance newsletters/information bulletins/websites	4.67 ± 0.594	0.13	4.29 ± 0.686	0.16	0.0133
1.3 Staffing					**0.1114**
1.3.1 Pharmacovigilance full/part-time staff inputs	5.00 ± 0.000	0.00	4.94 ± 0.243	0.05	0.0480
1.3.2 Pharmacovigilance staff duties established	5.00 ± 0.000	0.00	4.59 ± 0.795	0.17	0.0315
1.3.3 Personnel training	4.94 ± 0.236	0.05	4.76 ± 0.562	0.12	0.0319
2. Process					**0.3833**
2.1 Management and review					**0.1281**
2.1.1 Guidance on building pharmacovigilance systems in medical institutions	4.89 ± 0.323	0.07	4.65 ± 0.606	0.13	0.0472
2.1.2 Internal audit	4.50 ± 0.707	0.16	4.06 ± 0.827	0.20	0.0317
2.1.3 Sectoral cooperation	4.67 ± 0.485	0.10	4.29 ± 0.772	0.18	0.0278
2.1.4 data management	4.61 ± 0.502	0.11	4.41 ± 0.712	0.16	0.0214
2.2 ADR/ADE reporting and monitoring					**0.1401**
2.2.1 Collection and processing of individual security reports	5.00 ± 0.000	0.00	4.71 ± 0.588	0.12	0.0301
2.2.2 Quality review of reports	4.89 ± 0.471	0.10	4.65 ± 0.606	0.13	0.0270
2.2.3 Evaluation of reports	4.78 ± 0.548	0.11	4.88 ± 0.332	0.07	0.0280
2.2.4 Recording and transmitting drug safety information	4.72 ± 0.575	0.12	4.18 ± 0.809	0.19	0.0157
2.2.5 Serious/fatal/aggregate incident management	5.00 ± 0.000	0.00	4.88 ± 0.332	0.07	0.0393
2.3 Pharmaceutical risk management and feedback					**0.1150**
2.3.1 Risk signal management	4.83 ± 0.514	0.11	4.71 ± 0.686	0.15	0.0253
2.3.2 Risk management measures	4.78 ± 0.428	0.09	4.71 ± 0.588	0.12	0.0304
2.3.3 Issuance of drug safety bulletins	4.39 ± 0.850	0.19	4.29 ± 0.772	0.18	0.0212
2.3.4 Risk communication with stakeholders	4.56 ± 0.616	0.14	4.18 ± 0.636	0.15	0.0185
2.3.5 Regional sharing of pharmacovigilance data and survey results	4.67 ± 0.594	0.13	4.44 ± 0.629	0.14	0.0196
3. Outcome					**0.2636**
3.1 Effectiveness of adverse reaction monitoring					**0.1047**
3.1.1 Quality of ADR reporting	4.89 ± 0.323	0.07	4.88 ± 0.332	0.07	0.0442
3.1.2 Number of reported cases of adverse drug reaction per 1,000,000 people	4.28 ± 0.752	0.18	3.71 ± 0.985	0.27	0.0150
3.1.3 Percentage of new/serious adverse reaction reports	4.61 ± 0.698	0.15	4.65 ± 0.493	0.11	0.0285
3.1.4 Timeliness of adverse reaction reporting	4.83 ± 0.383	0.08	4.59 ± 0.618	0.13	0.0171
3.2 Number of regulatory actions					**0.0815**
Number of changes in safety information in drug instructions	4.44 ± 0.705	0.16	First round of Delphi deletion indicators		
Number of recalled drugs	4.11 ± 1.410	0.34	First round of Delphi deletion indicators		
3.2.1 Frequency of internal audits and impact analysis	4.44 ± 0.856	0.19	4.24 ± 0.664	0.16	0.0332
3.2.2 Number of risk signals recognized	First round of Delphi increased indicators		4.41 ± 0.712	0.16	0.0256
3.2.3 Number of training sessions for promotion	First round of Delphi increased indicators		4.47 ± 0.514	0.11	0.0227
3.3 Timeliness of communication and amount of feedback					**0.0774**
3.3.1 Percentage of feedback from higher authorities	4.11 ± 0.832	0.20	4.24 ± 0.752	0.18	0.0301
3.3.2 Timeliness of stakeholder feedback	4.11 ± 0.832	0.20	4.12 ± 0.697	0.17	0.0202
3.3.3 Timeliness of drug safety message response	4.61 ± 0.698	0.15	4.35 ± 0.702	0.16	0.0271

The bolded values refer to the weights of the primary and secondary indicators.

### 3.3 Weight distribution of the index

According to the AHP results, the weights of the primary indicators from high to low were as follows: process (0.3833), structure (0.3531), and results (0.2636). Among the secondary indicators, “Resource inputs” (0.1465) and “ADR/ADE Reporting and Monitoring” (0.1401) had higher weights, and the “Number of regulatory actions” (0.0815) and Timeliness of communication and amount of feedback” (0.0774) were lower. In addition, among the 35 tertiary indicators finally established, the top three in terms of weights were “Financial inputs” (0.0487), “Pharmacovigilance centers/departments/units” (0.0485), and “Pharmacovigilance full/part-time staff inputs” (0.0480) under the structure indicator. The majority of the tertiary indicators with relatively lower weights correspond to the outcome indicators (Table 3).

## 4 Discussion

This study develop an evaluation indicator system based on the WHO manual, relevant Chinese policies, and the current status of municipal-level drug safety surveillance in China. It has been successfully implemented by the Shaanxi Provincial Drug Regulatory Agencies to assess the pharmacovigilance systems in various municipal level cities. In practice, the system has demonstrated strong adaptability. It not only provides clear guidance for local regulatory authorities to improve the operation of pharmacovigilance systems but also lays a solid foundation for advancing and optimizing China’s drug safety regulatory framework.

This aspect is particularly significant for developing countries, where basic functionalities must first be established before progressing toward more advanced systems. Many developing countries face regulatory environments, technical resource limitations, and institutional constraints similar to those observed in Chinese municipal-level cities. For example, Garashi HY (Hussain and Hassali, 2019), in a qualitative study on the implementation of pharmacovigilance policies in Jordan, Oman, and Kuwait, found that these countries also encounter comparable challenges during system implementation, including insufficient systematic policy support, inadequate cross-departmental coordination, and underdeveloped communication and capacity-building mechanisms. Thus, this system not only serves as an effective evaluation tool for pharmacovigilance systems in China but also offers a feasible reference for developing countries in the early stages of constructing and operating pharmacovigilance systems.

To ensure the applicability and feasibility of the preliminary indicator system in the pharmacovigilance practice ofmunicipal-level cities in China, this study conducted a pre-survey in five selected cities during the framework development process. The study highlighted that performance evaluation should integrate both quantitative and qualitative dimensions. For example, in adverse drug reaction monitoring, in addition to quantitative metrics such as the “Number of reported ADR cases per 1,000,000 population” and the “Percentage of new or serious ADR reports”, qualitative indicators such as “Quality of ADR reporting” should also be incorporated to assess reporting performance in terms of completeness, accuracy, and timeliness. Meanwhile, this study further refined the categorization of dimensions under the main indicators, thereby providing a clearer depiction of the pharmacovigilance system’s operations across different levels.

Compared with the WHO core indicator system (see Supplementary File 3 for details), this study’s framework places greater emphasis on practicality and applicability, making it particularly suitable for nascent or resource-limited pharmacovigilance systems. For example, while the nine core process indicators proposed by WHO are representative, they impose relatively high demands for data collection and lack detailed operational guidance for individual workflow components. In contrast, the process indicator system developed in this study prioritizes practical implementation, covering sub-processes such as system establishment, internal auditing, cross-departmental collaboration, data management, along with ADR/AE report collection, quality review, risk signal management, and feedback. These components form a closed-loop workflow from reporting to risk communication, demonstrating greater refinement and operational feasibility, thereby better suiting regions with limited resources or those in the early stages of system development. At the same time, although the WHO manual considers the structure and functions of regulatory agencies, it does not adequately reflect the participation of stakeholders such as healthcare providers/facilities, public health programs, Marketing Authorization Holders (MAH), and consumers (Barry et al., 2020). Involvement of stakeholders in the policy formulation and implementation process has been identified as a crucial means to ensure effective pharmacovigilance implementation (Mwendera et al., 2019; Pan Canadian Joint Consortium for School Health, 2010). Based on this, the study incorporated stakeholder-related assessments into the indicator system. These include indicators focusing on the management and oversight of healthcare institutions, timely feedback of drug safety information to MAHs, and the dissemination of drug safety messages on public platforms.

Regarding the outcome dimension, WHO indicators focus more on overarching health impacts—such as drug-related hospitalization and mortality rates—which are often challenging to quantify in resource-limited settings. This study, however, emphasizes process outcomes and management performance, incorporating indicators that directly reflect routine output, including ADR report quality, timeliness of reporting, and timeliness of communication and amount of feedback. These indicators facilitate ongoing monitoring and evaluation, providing direct evidence for system optimization. Consequently, they are more appropriate for countries and regions where pharmacovigilance systems are still evolving, offering a step-by-step pathway for continuous improvement.

During our qualitative research on the implementation of pharmacovigilance policies, we came to the conclusion that policy support for conducting pharmacovigilance activities should consider policy goals and adapt to the current stage of institutional development (Garashi et al., 2021; Matland, 1995). Based on the Delphi expert consultation results, we have made appropriate adjustments to the indicator system. The implementation of pharmacovigilance activities relies on both policy support and institutional development to ensure that staff can carry out relevant work with specific authority. At the national level, there is usually a formulation of strategic plans, but at the provincial and municipal levels, specific plans need to be developed based on stated goals and delegated for implementation. This implies that even grassroots organizations should have corresponding “regulatory” authority to support personnel in exercising relevant responsibilities. Therefore, based on our expert recommendations, we added the “Establishment of Regulatory Framework” indicator.

At the same time, several adjustments were made to the outcome indicators during the first round of the Delphi process. Specifically, the items “number of drug recalls” and “number of modifications to drug instructions” were removed from the “regulatory actions” category, while “number of recognized risk signals” and “number of training sessions for promotion” were added. Experts explained that this adjustment was mainly based on two considerations. First, it reflects the institutional reality of China’s pharmacovigilance system. In China, the administrative authority to issue drug recall orders or approve safety-related revisions of drug labels resides primarily at the provincial regulatory level, whereas municipal pharmacovigilance centers mainly serve technical support and coordination functions. Therefore, using such regulatory indicators to evaluate the performance of municipal centers would be inappropriate, as these centers do not directly influence such regulatory decisions.

Secondly, there are limitations in the development stage. For regions still in the early stages of establishing a pharmacovigilance system, directly obtaining such indicator results is not only difficult in terms of data acquisition but also fails to accurately reflect their actual work performance. Consequently, experts recommended shifting the evaluation focus from “final outcomes” to “key processes,” emphasizing the implementation of specific measures that reflect risk management capacity. Among these, the “number of recognized risk signals” serves as a core output of pharmacovigilance activities and represents a feasible and meaningful performance indicator for municipal-level agencies under current conditions. The “number of training sessions for promotion” effectively captures the performance of municipal centers in guiding and training healthcare institutions and county-level monitoring sites, reflecting process-oriented achievements in capacity building. This adjustment makes the revised indicator system better reflect China’s policy context and regional realities, offering a more accurate and practical assessment of municipal pharmacovigilance performance.

During the two rounds of Delphi expert consultations, the indicator “Number of reported cases of adverse drug reaction per 1,000,000 people” generated considerable debate (CV > 0.25). Some experts noted that this indicator is easily affected by population mobility and uneven distribution of medical resources across regions. This is particularly evident in central cities, such as provincial capitals, which exert a siphoning effect on patients from surrounding areas, causing some adverse drug reactions to be reported outside the location where they occur. Other experts noted that despite its limitations, the indicator remains strategically important. It enables cross-regional comparisons adjusted for population size, aligns with national pharmacovigilance policies, and guides local efforts and resource allocation. As a core metric in system design, it was retained to ensure both policy compliance and practical relevance. It is noteworthy that in the subsequent AHP weighting analysis, this indicator was assigned a relatively low weight (0.015), reflecting a balance between “policy compliance” and “practical controversy”. While respecting the mandatory national evaluation requirements, its influence on the overall assessment is effectively moderated, ensuring that more stable and performance-reflective key indicators dominate the framework. This approach enhances both the scientific rigor and practical applicability of the evaluation system.

This study found that process indicators, with a weight of 0.3833, are the highest priority within the SPO framework. This suggests that in primary pharmacovigilance systems, strengthening process management and implementation may be the most direct and feasible way to improve drug safety management performance and enhance monitoring outcomes. Among third-level indicators under the structure category (0.3531), financial inputs (0.0487) and pharmacovigilance staffing (0.0480) are ranked highest. A country’s financial support for its pharmacovigilance system is directly related to drug safety measures, and budget constraints constitute one of the reasons why pharmacovigilance systems in middle- and low-income countries may not function properly (Khan et al., 2023; Ampadu et al., 2018). Financial investment supports the implementation of activities, training, and system improvements to ensure efficient data management and analysis. The allocation of professional personnel ensures the effective operation of the system, including quality assurance, timely communication, and emergency response. Together, these two aspects guarantee that the pharmacovigilance system is reliable and effective in identifying, monitoring, and responding to adverse drug events, thereby safeguarding patient safety. Although the WHO emphasizes the importance of drug regulation and information communication, tertiary indicators under the secondary indicators “Number of Regulatory Actions” and “Timeliness and Feedback of Information Communication” carry relatively lower weights. This may reflect that, at China’s current stage of nascent pharmacovigilance, awareness of regulation and information sharing still needs strengthening, which aligns with the present national context.

It should be noted that, under resource-limited conditions, the simultaneous optimization of structural and process elements is crucial for enhancing the overall performance of a pharmacovigilance system. Even with well-developed process management, the system’s effectiveness cannot be fully realized without structural support, such as financial investment, professional staffing, and infrastructure development. The relatively low weights of regulatory and communication indicators reflect that China’s pharmacovigilance system is currently in a phase where foundational construction and process optimization are progressing in parallel. Overall, the findings suggest that a pharmacovigilance system built on sufficient financial and human resources, complemented by efficient operational processes, represents a key pathway for establishing a sustainable and responsive system—particularly suitable for policy environments in developing countries facing challenges similar to those in China.

This study has several limitations that affect both the generalizability and the applicability of the proposed framework. First, due to time and resource constraints, the preliminary survey covered only five prefecture-level cities, which, despite being representative, may limit the accuracy of indicator prioritization and weighting. Second, in China’s regulatory system, prefecture-level pharmacovigilance institutions mainly perform technical and operational functions under provincial authorities and lack independent policy-making power—an institutional feature that shaped the framework’s design. As a result, the proposed indicator system primarily focuses on technical implementation, coordination, and performance monitoring within a centralized regulatory structure. While it provides a practical tool for assessing China’s pharmacovigilance capacity, its applicability in more decentralized governance contexts may be limited. Future research should adapt and test the framework across different governance models to improve its generalizability and comparative value.

## 5 Conclusion

In summary, this study develops an indicator framework model for evaluating China’s emerging pharmacovigilance system, based on extensive domestic and international literature and policy guidance. While the framework is developed from the Chinese context, it provides a theoretical foundation and a practical evaluation tool that may be adapted for use in regions with similar pharmacovigilance system maturity, contributing to more consistent practices and enhanced drug safety.

## Data Availability

The original contributions presented in the study are included in the article/Supplementary Material, further inquiries can be directed to the corresponding author.
