# Supplementary material for: Constructing an evaluation framework for primary pharmacovigilance systems in developing countries: a case study from China
Source: Front Pharmacol. 2025 Nov 18;16:1657574. doi: 10.3389/fphar.2025.1657574 (PMC12669157; doi:10.3389/fphar.2025.1657574)
Supplement: Supplementary file 1 [file Supplementaryfile1.docx]

Delphi survey questionnaire

Dear Expert,

Hello! First and foremost, we extend our deepest gratitude for your participation in this project consultation!

Our team is currently conducting a study titled "Constructing an Evaluation Framework for Primary Pharmacovigilance Systems in Developing Countries: A Case Study of China." The purpose of this study is to design an evaluation framework suitable for assessing the implementation and effectiveness of pharmacovigilance activities in various municipal level pharmacovigilance centers. By regularly evaluating the operation and current status of pharmacovigilance systems using this framework, we aim to identify deficiencies promptly and make necessary improvements. This will ensure the effective operation of pharmacovigilance activities, reduce drug safety risks, guide rational drug use, and promote the development of the healthcare sector, thereby maximizing the safety of medication for the public.

Based on a literature review and Donabedian's three-dimensional theory: structure-process-outcome, we initially divided the pharmacovigilance evaluation framework into three dimensions, which constitute the primary indicators of the system. We then referred to the "WHO Pharmacovigilance Indicators: A Practical Manual for the Assessment of Pharmacovigilance Systems", the WHO Global Benchmarking Tool (GBT) for pharmacovigilance, and previous reviews of laws, regulations, and guidelines. We also conducted one-on-one interviews with four experts from provincial drug regulatory departments to preliminarily draft nine secondary indicators and 34 tertiary indicators suitable for assessing the current status of pharmacovigilance systems in various municipal level centers.

To further refine this evaluation framework and enhance its suitability for long-term practical application and performance assessment, we are conducting this expert consultation. Given your extensive knowledge and rich experience in drug adverse reaction monitoring, pharmacovigilance-related research, and practical management, we sincerely invite you to serve as a consultation expert for this study.

This study requires two rounds of expert consultation surveys. This is the first round, and we kindly ask all experts to provide valuable feedback on the preliminarily established evaluation framework. After this consultation, we will compile and analyze your feedback and provide it in the next round of the consultation questionnaire, which will serve as a reference for further suggestions on the study content. To ensure the progress of the study, we hope you can respond to the questionnaire within one week. If you have any questions while completing the questionnaire, please feel free to contact us. Thank you for your support of this study. We wish you good health and a pleasant work experience!

March 7, 2023

Contact persons:

Shuang Lei (Email: ls1226343190@163.com)

Bianling Feng (Email: [fengbianling@163.com](mailto:fengbianling@163.com))

**一、Basic Information**

1. Basic personal information

Name：__________ Age：______ Education：______ Specialization：_________ Contact phone number：______

2. Basic work information

Work unit: ___________ Work department: ____________ Position: ________ Title: __________

3. Nature of work and years of experience

Research direction of your work: _______________ Years of working in this research direction: ______

**二、Indicator Scoring**

Instructions for Completing the Form: Below are the evaluation indicators within the pharmacovigilance assessment framework. The importance of the indicators represents the degree of importance of the indicator, and the feasibility of the indicator represents the ease of obtaining/building the indicator. Both are scored on a scale of 1-5.

Scoring standards for importance:

5 = Very Important

4 = Quite Important

3 = Moderately Important

2 = Slightly Important

1 = Not Important

Scoring standards for feasibility:

5 = Very Easy

4 = Quite Easy

3 = Moderately Easy

2 = Slightly Easy

1 = Not Easy

Please assess the relative importance and feasibility of each indicator and assign a suitable score to the corresponding option. If you believe the description of an indicator is inaccurate/unnecessary/unimportant (i.e., you score it as 1 or 2) or have other comments/suggestions, please fill in the modification/other comments section (you may attach additional documents for supplementary opinions). Also, if you think an indicator has poor feasibility (i.e., you score it as 1 or 2), you should explain the reasons.

Before making your evaluation, you may read the meanings of each indicator in Supplementary File 2 to understand our preliminary set opinions on the evaluation indicators.

（1）Scoring of Level 1 Indicators

| Primary indicators | importance rating | | | | | feasibility rating | | | | | revised opinion |
| --- | --- | --- | --- | --- | --- | --- | --- | --- | --- | --- | --- |
|  | 1 | 2 | 3 | 4 | 5 | 1 | 2 | 3 | 4 | 5 |  |
| 1. Structural |  | | | | |  | | | | |  |
| 2. Process |  | | | | |  | | | | |  |
| 3. Outcome |  | | | | |  | | | | |  |
| ***Complementary items*** |  | | | | |  | | | | |  |

（2）Scoring of secondary indicators

| Secondary indicators | | importance rating | | | | | feasibility rating | | | | | revised opinion | |
| --- | --- | --- | --- | --- | --- | --- | --- | --- | --- | --- | --- | --- | --- |
|  |  | 1 | 2 | 3 | 4 | 5 | 1 | 2 | 3 | 4 | 5 |  |  |
| 1. Structural | 1.1 Organization |  | | | | |  | | | | | *Under this indicator, it is proposed to add “Establishment of Regulatory Framework”* | |
|  | 1.2 Resource inputs |  | | | | |  | | | | |  | |
|  | 1.3 Staffing |  | | | | |  | | | | |  | |
| 2.Process | 2.1 Management and review |  | | | | |  | | | | |  | |
|  | 2.2 ADR/ADE Reporting and Monitoring |  | | | | |  | | | | |  | |
|  | 2.3 Pharmaceutical risk management and feedback |  | | | | |  | | | | |  | |
| 3.Outcone | 3.1 Effectiveness of Adverse Reaction Monitoring |  | | | | |  | | | | |  | |
|  | 3.2 Number of regulatory actions |  | | | | |  | | | | | *Under this indicator, it is proposed to add “Number of risk signals recognized”、“Number of training sessions for promotion”* | |
|  | 3.3 Timeliness of communication and amount of feedback |  | | | | |  | | | | |  | |
| ***Complementary items*** |  |  | | | | |  | | | | |  | |

（3）Scoring of the three levels of indicators

| Tertiary indicators | | importance rating | | | | | feasibility rating | | | | | revised opinion |
| --- | --- | --- | --- | --- | --- | --- | --- | --- | --- | --- | --- | --- |
|  |  | 1 | 2 | 3 | 4 | 5 | 1 | 2 | 3 | 4 | 5 |  |
| 1.1 Organization | 1.1.1 Pharmacovigilance centers/departments/units |  | | | | |  | | | | |  |
|  | 1.1.2 Advisory Board of Experts |  | | | | |  | | | | |  |
| 1.2 Resource inputs | 1.2.1 Financial inputs |  | | | | |  | | | | |  |
|  | 1.2.2 Pharmacovigilance information system |  | | | | |  | | | | |  |
|  | 1.2.3 Pharmacovigilance information resources |  | | | | |  | | | | |  |
|  | 1.2.4 Pharmacovigilance information completion tool |  | | | | |  | | | | |  |
|  | 1.2.5 Dissemination of pharmacovigilance newsletters/information bulletins/websites |  | | | | |  | | | | |  |
| 1.3 Staffing | 1.3.1 Pharmacovigilance full/part-time staff inputs |  | | | | |  | | | | |  |
|  | 1.3.2 Pharmacovigilance staff duties established |  | | | | |  | | | | |  |
|  | 1.3.3 Personnel training |  | | | | |  | | | | |  |
| 2.1 Management and review | 2.1.1 Guidance on building pharmacovigilance systems in medical institutions |  | | | | |  | | | | |  |
|  | 2.1.2 Internal audit |  | | | | |  | | | | |  |
|  | 2.1.3 Sectoral cooperation |  | | | | |  | | | | |  |
|  | 2.1.4 data management |  | | | | |  | | | | |  |
| 2.2 ADR/ADE Reporting and Monitoring | 2.2.1 Collection and processing of individual security reports |  | | | | |  | | | | |  |
|  | 2.2.2 Quality review of reports |  | | | | |  | | | | |  |
|  | 2.2.3 Evaluation of reports |  | | | | |  | | | | |  |
|  | 2.2.4 Recording and transmitting drug safety information |  | | | | |  | | | | |  |
|  | 2.2.5 Serious/fatal/aggregate incident management |  | | | | |  | | | | |  |
| 2.3 Pharmaceutical risk management and feedback | 2.3.1 Risk signal management |  | | | | |  | | | | |  |
|  | 2.3.2 Risk management measures |  | | | | |  | | | | |  |
|  | 2.3.3 Issuance of drug safety bulletins |  | | | | |  | | | | |  |
|  | 2.3.4 Risk communication with stakeholders |  | | | | |  | | | | |  |
|  | 2.3.5 Regional sharing of pharmacovigilance data and survey results |  | | | | |  | | | | |  |
| 3.1 Effectiveness of Adverse Reaction Monitoring | 3.1.1 Quality of ADR reporting |  | | | | |  | | | | |  |
|  | 3.1.2 Number of reported cases of adverse drug reaction per 1,000,000 people |  | | | | |  | | | | |  |
|  | 3.1.3 Percentage of new/serious adverse reaction reports |  | | | | |  | | | | |  |
|  | 3.1.4 Timeliness of adverse reaction reporting |  | | | | |  | | | | |  |
| 3.2 Number of regulatory actions | 3.2.1 Frequency of internal audits and impact analysis |  | | | | |  | | | | | *Recommended for deletion, the department has no authority to interfere with this activity* |
|  | 3.2.2 Number of risk signals recognized |  | | | | |  | | | | | *Recommended for deletion, the department has no authority to interfere with this activity* |
|  | 3.2.3 Number of training sessions for promotion |  | | | | |  | | | | |  |
| 3.3 Timeliness of communication and amount of feedback | 3.3.1 Percentage of feedback from higher authorities |  | | | | |  | | | | |  |
|  | 3.3.2 Timeliness of stakeholder feedback |  | | | | |  | | | | |  |
|  | 3.3.3 Timeliness of drug safety message response |  | | | | |  | | | | |  |
| *Complementary items* |  |  | | | | |  | | | | |  |

**Other relevant suggestions (other suggestions for adding/deleting/modifying indicators, if needed):**

| **Suggestions:** |
| --- |

**三、Quantitative Assessment of Expert Authority**

In Table 3-1, please mark "√" in the corresponding blank based on your familiarity with the evaluation criteria.

In Table 3-2, when assessing the criteria, judgments are typically influenced to varying degrees by the following four factors: theoretical analysis, practical experience, understanding of domestic and foreign peers, and intuition. Please mark "√" in the corresponding blank based on the influence level of these four aspects when making judgments.

Table 3-1 Expert Familiarity Level Table

| Familiarity Level | Very Familiar | Familiar | Moderately Familiar | Not Very Familiar | Unfamiliar |
| --- | --- | --- | --- | --- | --- |
| Expert Self-Assessment |  |  |  |  |  |

Table 3-2 Quantitative Assessment of Factors Influencing Expert Judgments

| Judging Criterion | High | Medium | Low |
| --- | --- | --- | --- |
| Theoretical Analysis |  |  |  |
| Practical Experience |  |  |  |
| Understanding of Peers (Domestic and Foreign) |  |  |  |
| Intuition |  |  |  |
