# Supplementary material for: Constructing an evaluation framework for primary pharmacovigilance systems in developing countries: a case study from China
Source: Front Pharmacol. 2025 Nov 18;16:1657574. doi: 10.3389/fphar.2025.1657574 (PMC12669157; doi:10.3389/fphar.2025.1657574)
Supplement: Supplementary file 2 [file Supplementaryfile2.docx]

**Supplementary File 2: Explanation of indicators for each level of assessment**

Table S1. Interpretation of primary indicators

| primary indicators | Interpretation of indicators |
| --- | --- |
| 1. Structural | Structure indicators assess the critical pharmacovigilance structures, systems, and mechanisms within the research context. The accessibility of infrastructure is required to support the implementation of pharmacovigilance activities, and structure indicators evaluate the visual elements of pharmacovigilance. Additionally, structure indicators assess the policies and regulatory frameworks that support the implementation of pharmacovigilance activities. |
| 2. Process | Process indicators assess the extent of pharmacovigilance activities, focusing on a series of activities that describe the pharmacovigilance mechanism. This includes the collection, organization, analysis, and evaluation of ADR/ADE reports. Other activities that directly or indirectly impact the aforementioned processes are also considered, evaluating the overall functioning of the pharmacovigilance system |
| 3. Outcome | Results and impact indicators measure the effectiveness of pharmacovigilance activities, assessing the outcomes and changes. This involves measuring the achievement of pharmacovigilance goals and evaluating the performance of related activities. It goes beyond quantitative aspects, also presenting the quality of outcomes, such as the quality of adverse reaction reports. |

**Note: Bolded and italicized are the first round of indicator-specific modifications**

Table S2. Interpretation of secondary indicators

| secondary indicators | | Interpretation of indicators |
| --- | --- | --- |
| 1.Structural | 1.1 Organization | The objective of this indicator is to ensure that the entity responsible for pharmacovigilance activities implements effective organizational and management practices. Relevant departments should have clear delineation of responsibilities and regulations. Effective organization and practices contribute to the efficient and effective conduct of pharmacovigilance activities. |
|  | 1.2 Resource inputs | The objective of this indicator is to ensure financial investment and support for the infrastructure and related hardware and software necessary for conducting pharmacovigilance activities. This will ensure the proper execution of the essential components of pharmacovigilance activities. |
|  | 1.3 Staffing | The objective of this indicator is to ensure that all activities related to pharmacovigilance within relevant departments are staffed with well-trained, experienced, and technically proficient personnel, ensuring that these personnel are empowered to fully perform their designated responsibilities. This will ensure that the pharmacovigilance process and activities are conducted according to best practices. This indicator is assessed by evaluating the human resource capabilities of each activity, including the number and composition of personnel, skills and experience, and specialized knowledge in specific areas required to fulfill pharmacovigilance functions. |
| 2. Process | 2.1 Management and review | The objective of this indicator is to ensure the execution of activities required for the pharmacovigilance system through unified management and standard procedures, including work instructions (such as data storage and regular internal audits). It aims to investigate the extent to which regulations and procedures governing the execution of various activities are well-established, ensuring the consistency, effectiveness, efficiency, fairness, and proportionality of the vigilance system. |
|  | 2.2 ADR/ADE Reporting and Monitoring | The establishment of this indicator, one of the main components of pharmacovigilance, focuses on Adverse Drug Reaction/Adverse Event (ADR/ADE) reporting and monitoring. Its goal is to ensure the consistent and efficient execution of ADR/ADE collection, processing, assessment, and reporting activities through unified management and standard procedures. This indicator explores the extent to which centers implement ADR/ADE reporting and monitoring activities to achieve consistency and maximize efficiency in their work. |
|  | 2.3 Pharmaceutical risk management and feedback | The goal of this indicator is to ensure that relevant departments carry out activities related to pharmaceutical risk management through unified management, standard procedures, and work instructions. This includes coordinating with higher-level authorities to implement required risk management measures for pharmaceutical risks and, based on this foundation, communicating and providing feedback to stakeholders in a timely manner. |
| 3. Outcome | 3.1 Effectiveness of Adverse Reaction Monitoring | The objective of this indicator is to ensure the effectiveness of pharmaceutical-related surveillance activities, primarily through performance evaluation of the collection and reporting of Adverse Drug Reactions (ADR/AE). This includes, but is not limited to, provincial centers assigning score indicators to various prefecture-level cities, assessing relevant progress outcomes, to ensure the effectiveness of activities in various prefecture-level cities. |
|  | 3.2 Number of regulatory actions | This indicator measures the outcomes of relevant departments taking proactive or collaborative risk management measures as directed by higher authorities. It assesses the regulatory decisions made based on pharmaceutical surveillance activities to ensure the safe use of drugs in that environment. It also evaluates the functionality of the Pharmacovigilance (PV) Center and the alignment between the activities of the PV Center and regulatory agencies. |
|  | 3.3 Timeliness of communication and amount of feedback | This indicator assesses the internal communication, transparency, and connections with the public and regional partners within the pharmacovigilance departments of various prefecture-level cities. It evaluates whether there is close communication and collaboration, aiding mutual understanding and engagement with all stakeholders involved in the pharmacovigilance system. This contributes to building confidence in the regulatory system. |

Table S3. Interpretation of tertiary indicators

Table S3-1. Interpretation of structural indicators under the tertiary indicators

| secondary indicators | tertiary indicators | Interpretation of indicators |
| --- | --- | --- |
| 1.1 Organization | 1.1.1 Pharmacovigilance centers/departments/units | This indicator indicates the presence of a pharmacovigilance center, department, or unit with a standard office space. The unit should have clearly defined responsibilities for conducting pharmacovigilance activities/adverse reaction monitoring. Note that this center should not include non-functional pharmacovigilance centers or those in the developmental non-delegated stage. |
|  | 1.1.2 Advisory Board of Experts | This refers to the existence of a qualified committee that can provide consultation and technical assistance on causality assessment, risk assessment, risk management, case investigation, and crisis communication when necessary. |
|  | ***1.1.3*** ***Establishment of Regulatory Framework***  ***(First round of Delphi Increased Indicators)*** | ***This indicator highlights the commitment of governmental institutions to ensuring the safe use of drugs, allowing operators to carry out their work with confidence. It clarifies the specific responsibilities and roles of the authorized authorities in conducting pharmacovigilance activities.*** |
| 1.2 Resource inputs | 1.2.1 Financial inputs | Financial arrangements for the pharmacovigilance center refer to providing regular (e.g., annual) and sustained funding sources to ensure the facility's operation, indicating the center's capability to conduct pharmacovigilance activities in that environment. The entities providing financial support include provincial center contributions, municipal financial allocations, and institutional financial inputs (including but not limited to regular/specialized financial allocations). |
|  | 1.2.2 Pharmacovigilance information system | The center should be equipped with corresponding devices and resources to meet the needs of pharmacovigilance work. Information technology systems are essential tools for conducting pharmacovigilance activities and should include:  1. Hardware facilities to support pharmacovigilance activities (computers, networks, filing cabinets, office space, printing equipment, etc.).  2. Software facilities to support pharmacovigilance activities (such as adverse reaction reporting systems). |
|  | 1.2.3 Pharmacovigilance information resources | The personnel responsible for various vigilance activities, including members of any expert committees, should have access to internal and external sources of information and reference materials. These materials should include:  1. Resources databases to support pharmacovigilance activities (literature search resources, medical dictionaries, adverse reaction databases, etc.), enabling them to make decisions about vigilance events.  2. Information resources, among other things, should encompass all scientific information related to drug use and its outcomes (i.e., quality, non-clinical, and clinical data, including pharmacovigilance and pharmacoepidemiological data). Access to market information may also be helpful. |
|  | 1.2.4 Pharmacovigilance information completion tool | This indicator measures the presence of data collection tools in the setup of pharmacovigilance operations. It indicates that the necessary tools for collecting key information on suspected cases of drug-related harm have been fully incorporated into the pharmacovigilance system. The reporting forms should include all the necessary elements for conducting a causality assessment based on clinical evidence. A review should be conducted to determine whether there is a unified standard for these forms in the region. |
|  | 1.2.5 Dissemination of pharmacovigilance newsletters/information bulletins/websites | This indicator refers to the existence of a system for regularly disseminating drug safety information to relevant professionals and the public (communication/information bulletins/websites/public accounts). Regular communication during crises and the strategic communication are among the minimum requirements for the normal operation of the pharmacovigilance system. |
| 1.3 Staffing | 1.3.1 Pharmacovigilance full/part-time staff inputs | This indicator explores the input of pharmacovigilance personnel in terms of both capability and quantity. It examines whether the center allocates a sufficient number of competent personnel (i.e., in terms of education, training, skills, and experience) to carry out pharmacovigilance activities. It should verify that the assigned human resources for pharmacovigilance activities are adequate in terms of quantity and are competent in necessary skills, education, experience, and training. |
|  | 1.3.2 Pharmacovigilance staff duties established | This indicator refers to whether the pharmacovigilance personnel's work procedures are in place, including documented procedures for job responsibilities and workflows. It examines whether relevant duty regulations are established, and job descriptions should outline the responsibilities, duties, and necessary capabilities of the current staff. |
|  | 1.3.3 Personnel training | The center should provide training for relevant staff, with the training content tailored to different levels of pharmacovigilance knowledge and skills corresponding to different positions and responsibilities. The development and implementation of personnel training plans should be included in quality control indicators, and the number of training sessions should also comply with relevant regulations. |

**Note: Bolded and italicized are the first round of indicator-specific modifications**

Table S3-2. Interpretation of process indicators under the tertiary indicators

| secondary indicators | tertiary indicators | Interpretation of indicators |
| --- | --- | --- |
| 2.1 Management and review | 2.1.1 Guidance on building pharmacovigilance systems in medical institutions | Local drug regulatory authorities should, in consideration of the actual regulatory situation in their administrative regions, incorporate relevant pharmacovigilance inspection content into routine regulatory work. They should scientifically formulate inspection plans, organize and implement them in an orderly and efficient manner. In the course of their work, they can further refine related tasks and enhance requirements. They should effectively fulfill local regulatory responsibilities. Specific guidance for the establishment of pharmacovigilance systems in healthcare institutions should also be developed. For example:  1. Verify that healthcare institutions have established relevant processes applicable to pharmacovigilance activities, with comprehensive and clear content.  2. Conduct regular/timely inspections of the work of healthcare institutions in the region and maintain records (e.g., check and provide timely feedback and guidance on the quality of ADR/ADE reports from healthcare institutions).  3. Provide feedback on areas where there are issues in the pharmacovigilance system construction of healthcare institutions in the region (e.g., guide healthcare institutions in using ADR/ADE reporting systems and regularly maintain them).  4. Conduct regular pharmacovigilance-related knowledge training for healthcare institutions. |
|  | 2.1.2 Internal audit | Regular evaluations of the implementation and operational effectiveness of the pharmacovigilance system should be conducted, establishing a dynamic adjustment mechanism to gradually improve the pharmacovigilance system. For example:  1. The center should develop internal audit SOPs/audit plans, which should include the objectives, scope, methods, standards, audit personnel, audit records, and reporting requirements for internal audits. The development of audit plans should consider key activities and positions in pharmacovigilance, as well as previous audit results, to assess whether the internal audit plan is independent, systematic, and comprehensive.  2. Actively (rather than passively) discuss the internal collection of ADR reports and issues related to report quality within the department on a regular/timely basis, and take appropriate measures based on the results of these discussions.  3. Audit changes in laws, regulations, and guidelines and promptly discuss and formulate relevant supporting implementation plans.  4. Personnel/function changes that may affect pharmacovigilance activities need to be reviewed and discussed promptly. |
|  | 2.1.3 Sectoral cooperation | Provide the necessary expertise and relevant recommendations to relevant departments for public health planning, new drug registration and market approval (NDA), prescription-to-OTC switch evaluations, etc. Good collaboration between departments is conducive to ensuring the safety of the entire drug life cycle. |
|  | 2.1.4 data management | The management process of relevant documents generated during the execution of pharmacovigilance activities, and strict adherence to pharmacovigilance document archiving, including the handling of individual safety reports and preservation of original data, should encompass:  1. Whether key pharmacovigilance activities are documented.  2. The authenticity and accuracy of the recorded data.  3. Completeness and traceability of records and data.  4. Clarity and readability of handwritten records, preventing easy erasure.  5. Establishment of operational procedures, regular backups, and permission settings in the electronic record system.  6. Traceability and audit trails for data modifications.  7. Measures to ensure the security, confidentiality, and integrity of records and data, preventing damage or loss. |
| 2.2 ADR/ADE Reporting and Monitoring | 2.2.1 Collection and processing of individual security reports | Collect adverse drug reactions/adverse events of drugs based on standard procedures. Collection channels should include healthcare institutions, literature searches, public reports, pharmaceutical business enterprises, etc. Additionally, maintain records of feedback provided for non-compliant and inadequate reports. |
|  | 2.2.2 Quality review of reports | Timely/regularly assess the quality of reports (including completeness and timeliness). Provide prompt feedback to relevant stakeholders for inadequate reports. The content of report quality review should include:  1. Whether different reports are submitted within the specified time frame.  2. Whether the completeness of reports meets policy requirements (should include at least four elements: identifiable patient, identifiable reporter, suspected drug, drug adverse reaction).  3. Whether original records and follow-up records are traceable. |
|  | 2.2.3 Evaluation of reports | Evaluate reports of qualified adverse drug reactions/adverse events based on standard procedures, including assessments of causality, severity, and novelty/commonality. |
|  | 2.2.4 Recording and transmitting drug safety information | Record compliant reports and submit them to the superior monitoring center as required. Provide timely feedback to relevant stakeholders for non-compliant reports. |
|  | 2.2.5 Serious/fatal/aggregate incident management | A standing advisory committee or other expert group should handle, investigate, evaluate, and analyze severe/death/clustered events. Establish standardized procedural documents, for example, city and county-level centers should assist relevant departments in investigating adverse events related to drug groups. Cooperate with higher-level monitoring agencies to complete the review and handling of severe/death events, such as emergency response (investigation mechanism) for signals/alerts of clustered adverse events, urgent events, and death cases related to drug adverse events, as well as follow-up mechanisms for serious adverse reaction reports and unexpected adverse reactions. |
| 2.3 Pharmaceutical risk management and feedback | 2.3.1 Risk signal management | The signal management process includes signal detection, signal validation, signal analysis and prioritization, signal evaluation, and action recommendations, specifically:  1. Municipal-level centers should have a workflow for risk signal mining (the process of finding or identifying signals using all available sources) to promptly discover relevant safety signals (signal detection).  2. Municipal-level centers should further validate the relevance of potential signals discovered from various data sources (signal validation).  3. Prioritize the evaluation of events affecting the public, severe adverse events, adverse events in high-risk populations, and other specified relevant circumstances (signal analysis and prioritization).  4. The center should comprehensively summarize relevant information, assess the detected signals, make an integrated judgment on whether the signals constitute new drug safety risks, with three possible outcomes: signal uncertainty, signal negation, and signal affirmation.  5. Action recommendations (based on the evaluation results, provide suggestions for measures to be taken, such as continuous monitoring for signals with uncertainty). |
|  | 2.3.2 Risk management measures | For identified risk signals, relevant risk management measures should be taken to reduce drug safety risks. Municipal-level centers should cooperate with the risk minimization measures issued by higher-level authorities to better reduce drug safety risks and guide clinical medication. Risk minimization measures include routine and additional measures. Routine measures are similar to updating the product information. Additional measures typically involve risk communication (communication with other institutions in the region/signal communication with healthcare institutions or companies), educational programs, patient diaries, prescription restriction programs, controlled distribution, disease/drug registration recruitment programs, contraceptive programs, etc. |
|  | 2.3.3 Issuance of drug safety bulletins | There are appropriate mechanisms available and established to provide regular feedback and information to the public (including patients, parents, and caregivers) about the surveillance system, especially for serious events and outbreaks. In addition, providing guidance to the public on methods and approaches to managing any potential risks may significantly contribute to reducing or eliminating risks. Reports of investigations of public concern and summaries of these reports should be made available to the public. Regular publications or awareness-raising meetings will serve to evaluate and review the effectiveness of the communication mechanisms, as requested by and reviewed by assessors. |
|  | 2.3.4Risk communication with stakeholder | There are appropriate mechanisms available and established to facilitate the sharing of information about the surveillance system among all stakeholders involved in pharmacovigilance, especially for serious events and clusters. |
|  | 2.3.5 Regional sharing of pharmacovigilance data and survey results | The sharing and exchange of national alert data, investigation results, and conclusions with relevant regional partners (such as other prefecture-level cities) should be verified, and timely communication with regional partners about noteworthy risk signals discovered in routine work is essential. |

Table S3-3. Interpretation of outcome indicators under the tertiary indicators

| secondary indicators | tertiary indicators | Interpretation of indicators |
| --- | --- | --- |
| 3.1 Effectiveness of Adverse Reaction Monitoring | 3.1.1 Quality of ADR reporting | Evaluate the quality of ADR/ADE reports submitted by prefecture-level cities based on the national standards for ADR/ADE report quality assessment. The final score serves as the measure of ADR/ADE report quality. |
|  | 3.1.2 Number of reported cases of adverse drug reaction per 1,000,000 people | The average reporting quantity per million people is one of the key indicators for assessing the level of national drug adverse reaction monitoring. According to WHO requirements for ADR reporting, each country should have a minimum of 300 ADR reports per million people annually, with not less than 30% of them being severe cases. Comparing the ADR reporting per million population among prefecture-level city centers is beneficial for evaluating the level of drug adverse reaction monitoring in different regions, making it an important performance evaluation indicator. |
|  | 3.1.3 Percentage of new/serious adverse reaction reports | The proportion of new and severe drug adverse reaction/events reports is one of the important indicators for assessing the overall quality and utility of the reports. The monitoring and evaluation of drug adverse reactions have always focused on the collection and evaluation of new and severe reactions. An increase in their quantity indicates that regulatory authorities have a more comprehensive grasp of information, a better understanding of the risks associated with drugs, more manageable risks, a more evidence-based evaluation of drugs, and more accurate regulatory decisions. |
|  | 3.1.4 Timeliness of adverse reaction reporting | According to regulations, each prefecture-level city determines the types of ADR/ADE reports to be reported to higher-level authorities within the specified scope of events. |
| 3.2监管行动数量 | ***Number of changes in safety information in drug instructions(First round of Delphi deletion indicators)*** | ***The center should promptly notify and take action on drugs that require changes to the safety information in the package insert. Major situations for changes in drug package inserts, labels, etc., include:***  ***Revising the package insert in accordance with the requirements announced by the National Administration.***  ***Referring to the latest package insert for innovative drugs, improved new drugs, or reference formulations listed in the "Catalog of Generic Drug Reference Preparations" approved and marketed in China, and making changes to the safety information in the package insert for generic chemical drugs or biosimilar drugs.*** |
|  | ***Number of recalled drugs(First round of Delphi deletion indicators)*** | ***The number of drugs recalled due to safety issues as required in the risk management measures implemented by the center.*** |
|  | 3.2.1 Frequency of internal audits and impact analysis | According to the requirements of drug quality management, conduct periodic internal audits of the drug vigilance system and activities. Review the implementation of various system procedures, assess the appropriateness, completeness, and effectiveness of the drug vigilance system. Ensure that the monitoring center conducts regular analyses of the effectiveness of drug vigilance activities and takes appropriate measures based on the results to enhance the efficiency of the drug vigilance system. |
|  | ***3.2.2*** ***Number of risk signals recognized (First round of Delphi Increased Indicators)*** | ***Each center confirms risk signals based on the drug risk management process. The signal is considered effectively managed when it is eventually incorporated into the drug package insert modification plan or adopted by the National Medical Products Administration as part of other risk management measures, such as issuing drug safety announcements.*** |
|  | ***3.2.3*** ***Number of training sessions for promotion (First round of Delphi Increased Indicators)*** | ***China's policies require municipal-level relevant departments to undertake the promotion, training, and other tasks related to drug adverse reaction reporting and monitoring. Training should be provided to healthcare institutions and county-level adverse reaction monitoring points for drug vigilance and related activities. The frequency and duration of training sessions serve as performance indicators for such tasks.*** |
| 3.3 Timeliness of communication and amount of feedback | 3.3.1 Percentage of feedback from higher authorities | The number of inadequate reports provided as feedback by the higher-level monitoring center as a percentage of the total ADR/ADE reports submitted by that center in the previous calendar year. |
|  | 3.3.2 Timeliness of stakeholder feedback | It should be verified whether the center provides feedback to relevant stakeholders on drug safety information within the specified time frame, including feedback on the review of relevant content from healthcare institutions. |
|  | 3.3.3 Timeliness of drug safety message response | Pharmacovigilance alerts should be issued in a timely manner in response to domestic and international drug safety information, especially drug safety information and risk management measures (e.g., changes in instructions) issued at the national level, and local municipalities should respond in a timely manner by issuing drug safety information at the local level to ensure that it reaches stakeholders. |

**Note: Bolded and italicized are the first round of indicator-specific modifications**
